# Properties of Biocomposite Film Based on Whey Protein Isolate Filled with Nanocrystalline Cellulose from Pineapple Crown Leaf

**DOI:** 10.3390/polym13244278

**Published:** 2021-12-07

**Authors:** Fitriani Fitriani, Sri Aprilia, Nasrul Arahman, Muhammad Roil Bilad, Hazwani Suhaimi, Nurul Huda

**Affiliations:** 1 Doctoral Program, School of Engineering, Post Graduate Program, Universitas Syiah Kuala, Banda Aceh 23111, Indonesia; fitriani18@mhs.unsyiah.ac.id; 2 Department of Chemical Engineering, Universitas Syiah Kuala, Banda Aceh 23111, Indonesia; nasrular@unsyiah.ac.id; 3 Faculty of Integrated Technologies, Universiti Brunei Darussalam, Bandar Seri Begawan BE 1410, Brunei; hazwani.suhaimi@ubd.edu.bn; 4 Faculty of Food Science and Nutrition, Universiti Malaysia Sabah, Jalan UMS, Kota Kinabalu 88400, Sabah, Malaysia; drnurulhuda@ums.edu.my

**Keywords:** biocomposite film, whey protein isolate, nanocrystalline cellulose, pineapple crown leaf, film properties

## Abstract

Among the main bio-based polymer for food packaging materials, whey protein isolate (WPI) is one of the biopolymers that have excellent film-forming properties and are environmentally friendly. This study was performed to analyse the effect of various concentrations of bio-based nanocrystalline cellulose (NCC) extracted from pineapple crown leaf (PCL) on the properties of whey protein isolate (WPI) films using the solution casting technique. Six WPI films were fabricated with different loadings of NCC from 0 to 10 % *w*/*v*. The resulting films were characterised based on their mechanical, physical, chemical, and thermal properties. The results show that NCC loadings increased the thickness of the resulting films. The transparency of the films decreased at higher NCC loadings. The moisture content and moisture absorption of the films decreased with the presence of the NCC, being lower at higher NCC loadings. The water solubility of the films decreased from 92.2% for the pure WPI to 65.5% for the one containing 10 % *w*/*v* of NCC. The tensile strength of the films peaked at 7% NCC loading with the value of 5.1 MPa. Conversely, the trend of the elongation at break data was the opposite of the tensile strength. Moreover, the addition of NCC produced a slight effect of NCC in FTIR spectra of the WPI films using principal component analysis. NCC loading enhanced the thermal stability of the WPI films, as shown by an increase in the glass transition temperature at higher NCC loadings. Moreover, the morphology of the films turned rougher and more heterogeneous with small particle aggregates in the presence of the NCC. Overall, the addition of NCC enhanced the water barrier and mechanical properties of the WPI films by incorporating the PCL-based NCC as the filler.

## 1. Introduction

Biodegradable materials for food packaging are increasingly focused on preserving quality, extending food shelf life, and being environmentally friendly [1]. The use of bio-based polymers for food packaging such as polysaccharides, lipids, and proteins is a promising solution to replace petroleum-derived polymers and, at the same time, utilise abundant raw materials in nature [2,3]. Some biopolymers, such as PLA, starch, chitosan, cellulose, and protein-based, are currently used and produced as biofilms in the packaging market [4]. This biopolymer packaging, which is derived directly from agricultural waste, can be further optimised by combining various nanoparticles, additives, and nutrients, such as antimicrobials, antioxidants, colouring agents to improve the properties of films [5,6]. Some of the development biopolymers as packaging materials with properties and applications that have been studied in recent years are shown in Table 1.

Whey protein isolate (WPI) is a bio-based material with high protein concentrations. It is isolated from milk by-products in the cheese and tofu manufacturing process. WPI has excellent film-forming and gas barrier properties compared to other petroleum-based polymers, as reported earlier [5,18,19,20]. However, WPI films are limited in their ability as packaging due to their poor performances as a moisture barrier and weak mechanical properties originating from a high content of hydrophilic amino acids [21,22,23,24]. WPI films can be reinforced or chemically modified to overcome the weakness by incorporating nanoscale materials into their structure, as explored in this study.

Research on cellulose from bioresources as reinforcing materials or fillers in polymeric matrices has rapidly grown. Cellulose materials with nanoscale crystal size and phase are preferred because of their environmental advantages and exceptional mechanical properties [25,26,27]. Among several methods and treatments to produce nanocrystalline cellulose (NCC), the acid hydrolysis method is the most common way to isolate cellulose from its fibres. In the hydrolysis process, the acid breaks down the amorphous segments of the cellulose and releases single crystals segments [25,28,29,30,31]. In terms of isolation by acid hydrolysis, the process conditions such as temperature, acid concentration, and hydrolysis time are essential in affecting the properties of the resulting NCC [26,28,32]. Most agricultural by-products such as rice straw [33], kenaf bast [34], sugarcane bagasse [35], bamboo [36], *Typha latifolia* [25], pineapple leaf [28], and corncob [37] have been studied as a resource for the production of NCC.

Pineapple is one of the main agricultural products. The pineapple processing produces waste in leaf and fruit skin, which causes environmental problems if not appropriately treated [26,28]. Generally, the crown leaf by-product is made up of 79–83% cellulose, 19% hemicellulose, and 5–15% lignin [8]. Therefore, pineapple crown leaf (PCL) can be considered a source of cellulose and NCC and further used as a filler for WPI films for packaging purposes. Few studies have used PCL as a source for the isolation of nanocrystalline cellulose as a filler [26]. Nonetheless, the use of NCC from PCL as a filler for biocomposite film has not been reported.

Studies have been carried out on WPI-based films reinforced with nanoparticles [14,24,38]. However, there is no traceable evidence of NCC as a reinforcing agent to produce WPI-based film for packaging. The objective of this study was to extract NCC from PCL and investigate its potential as the reinforcement agent or filler in the manufacture of composite and biodegradable WPI-based films. After fabrication, the effects of NCC on the physical, mechanical, morphology, and thermal properties of WPI-based biocomposite film were also investigated.

## 2. Materials and Methods

### 2.1. Materials

PCL waste as NCC resources was obtained from a local market in Banda Aceh, Indonesia. Sodium hydroxide (NaOH, Merk, Darmstadt, Germany), hydrogen peroxide (H_2_O_2_, Merck), and sulfuric acid (H_2_SO_4_, Merck) were used as alkali, bleaching, and hydrolysis agents, while WPI (90% protein, Glanbia Nutrionals, Kilkenny, Ireland) and glycerol (Merck) were used as biopolymer matrix and plasticiser.

### 2.2. Preparation of Nanocrystalline Cellulose from Pineapple Crown Leaf

The NCC was prepared by acid hydrolysis according to the procedure reported earlier [26]. Before removing the non-cellulosic component through the first treatments, the PCL was washed with warm water to remove some soluble impurities, then dried in an oven at 60 °C for 24 h. The dried PCL fibres were cut into small pieces before the subsequent treatment. The alkali and bleaching treatments of the PCL were performed by using NaOH and H_2_O_2_ 1 M at 80 °C for 1 h. After the process was complete, the samples were rinsed several times using distillate water until reaching a neutral pH. After the alkali and bleaching treatments, the samples were hydrolysed using H_2_SO_4_ 3M at 45 °C for 2 h. The reaction in acid hydrolysis was stopped by adding 500 mL of distilled water and cooling in a water bath for 24 h. The NCC suspension was washed, centrifugated (2000 rpm for 30 min), and ultrasonicated (30 min) to remove the excess acid until pH neutral was reached. Finally, the resultant product was grounded into powder after the drying process and was stored at room temperature until further usage.

### 2.3. Preparation of Biocomposite Film

**Figure 1:**
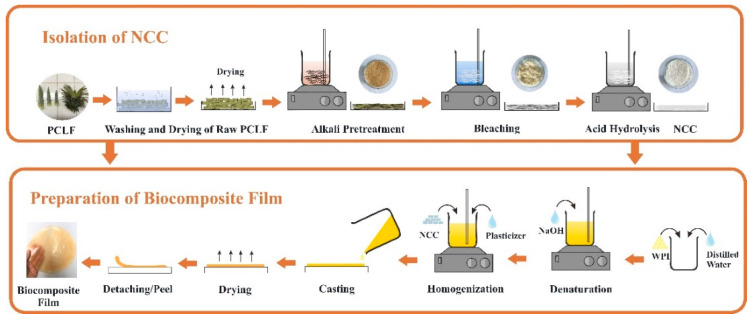
Schematic description of biocomposite film preparation.

WPI film solution with a concentration of 10% (*w*/*v*) was prepared in distilled water at pH neutral (regulated via step-wise addition of NaOH) at room temperature for 2 h to hydrate the proteins. Subsequently, the WPI solution was heated in a water bath at 90 °C for 30 min under constant stirring. Glycerol 6% (*w*/*v*) was added to the WPI solution before degassing to remove entrapped air bubbles at a temperature of 4 °C for 24 h. To prepare WPI/NCC biocomposite films, the solution of NCC was added in different ratios of 1, 3, 5, 7, and 10% (*w*/*w*) with 6% glycerol. The WPI and WPI/NCC solutions were poured and spread in a silicon mould with 20 cm × 15 cm, then dried in an oven at 60 °C for 24 h. The schematic illustrations of the NCC isolation and biocomposite film preparation are illustrated in Figure 1.

### 2.4. Physical and Mechanical Properties

The thickness of the resulting films was measured by using a micrometre with an accuracy of 0.001 mm at five random locations for each sample. The average value of thickness was used later in the calculation of the mechanical properties. The transparency of the biocomposite films was measured at wavelengths of 300–700 nm using a UV–Vis spectrophotometer (UV-1700, Shimadzu, Kyoto, Japan) according to ASTM D1746-09 standard. The moisture contents (MC) of the biocomposite films were measured from the weight loss of film specimens of 3 cm × 3 cm after drying in an oven at 105 °C. Measurements were performed with three replications for each film sample, and the MC was calculated using Equation (1) as follows:

(1) $$\mathrm{MC}\;\left(\%\right){=W}_{1}-W_{2}$$

where W_1_ and W_2_ are the weight loss of biocomposite films before and after drying, respectively.

The moisture absorption of the films was measured according to the modified procedure reported earlier [14,39]. Dry film specimens of 2 cm × 2 cm in 0% relative humidity (RH) were used as reference. The specimens were then placed into a desiccator to enhance RH to 55% until the equilibrium state was obtained. The moisture absorption was calculated using Equation (2) as follows:

(2) $$\mathrm{WS}\;\left(\%\right)=\frac{W_{t}-W_{0}}{W_{0}}\times 100$$

where W_0_ and W_t_ are the initial weight at 0% RH and the weight after equilibrium under 55% RH.

The films’ water solubility (WS, %) was determined as the soluble dry matter content ratio before and after immersion in water. The film specimen (3 cm × 3 cm) was cut and dried at 50 °C for 5 h in an oven, then weighed to measure the initial dry weight. Then, it was immersed in 40 mL of distilled water for 24 h at room temperature. Finally, the insoluble film was dried at 110 °C for 5 h in an oven to obtain the final weight. The WS was determined according to Equation (3).

(3) $$\mathrm{WS}\;\left(\%\right)=\frac{\mathrm{initial}\;\mathrm{dry}\;\mathrm{weight}-\mathrm{final}\;\mathrm{dry}\;\mathrm{weight}}{\mathrm{initial}\;\mathrm{dry}\;\mathrm{weight}}\times 100$$

The tensile strength (TS) and elongation at break (EB) of the films were measured using an MTS Exceed Universal Testing Machine E43 (China) according to ASTM standard method D638.

### 2.5. Morphology and Thermal Properties of Nanocrystalline Cellulose

The surface and cross-sectional morphology of the films were examined by scanning electron microscopy (SEM, JEOL JSM-6360OLA, Tokyo, Japan). The films were coated with a thin layer of gold and then observed under a voltage of 14 kV. The functional groups of the films were captured using a Fourier-transform infrared spectroscopy (FTIR) spectrophotometer (FTIR, IRPrestige-21, Shimadzu, Kyoto, Japan). The spectra of the films were gained with scanning ranges between 4000 to 400 cm^−1^ using 25 scans at a resolution of 4 cm^−1^ for each sample. To improve the result data of FTIR spectra, principal component analysis (PCA) was carried out using the OriginPro Software (OriginLab, Northampton, MA, USA). The numbers in PCA associated with the names of every sample in this analysis corresponded to the condition of FTIR measurements.

The thermal stability of the films was determined using Shimadzu DTG-60 (Kyoto, Japan) through thermogravimetric analysis (TGA). The sample films were heated from 25 to 500 °C with a heating rate of 10 °C min^−1^ under an inert atmosphere. Weight loss of the films was measured as a function of temperature.

### 2.6. Statistical Analysis

The experiments were conducted in triplicate in a completely randomised design, and the obtained data were further analysed statistically. Normality of distribution was tested by the Shapiro–Wilk test. One-way analysis of variance (ANOVA) and Duncan multiple range tests were used to determine the significant difference between treatments at a 95% (*p* < 0.05) confidence level using SPPS 25 (Version 25, SPSS Inc., Chicago, IL, USA) software.

## 3. Results and Discussion

### 3.1. Physical Properties

#### 3.1.1. Film Thickness

Film thickness is one of the parameters in physical properties that can be affected by the addition of NCC to the WPI biopolymer. Table 2 lists the thickness data of the films showing that they increased in the presence of NCC. The thickness of WPI pure film was 0.081 mm, while the thicknesses of WPI film with 1%, 3%, and 10% of NCC were 0.112, 0.117, and 0.091 mm. The WPI films containing NCC were significantly thicker than the pure WPI film (*p* < 0.05). The thicknesses variations are usually associated with the distribution of fillers and an increase in the solid content of the resulting films. These results are consistent with previous reports of protein isolate film with the addition of microfibrillated cellulose and oat-based NCC [14,40].

#### 3.1.2. Film Transparency

Film transparency is one of the essential characteristics of food packaging. It shows the ability of the polymer matrix to blend with fillers and can affect the appearance of the packaged products [14]. The transparency of WPI films ranged from 300 to 800 nm, which are summarised in Table 3. Based on the results, pure WPI film had the highest transparency, compared with the ones with the addition of NCC. The transparency of films decreased significantly as the concentrations of NCC increased (*p* < 0.05). This reduction was probably because of the light scattering of NCC particles that were distributed in the biopolymer network, which reduced the transmission of light [22].

Increasing NCC loading in the WPI films reduced the transparency from 15.8% (pure WPI) to 2.3% for the film containing 10% NCC at a wavelength of 800 nm (*p* < 0.05). The results obtained in this study agree with the previous reports [14,41], where the transparency of the WPI film decreased with the addition of nanoparticles fillers. In this study, the transparency of the obtained biocomposite film was still lower than the WPI film reported earlier [14]. In these cases, the biocomposite film from WPI loaded with NCC still required further improvements in the processing of the film solution.

#### 3.1.3. Moisture Content

The MC of a WPI film is associated with the hollow space occupied in the structural network by water molecules [14,42]. Table 4 shows that the MC decreased slightly with the increase in NCC concentration in the WPI films (*p* < 0.05). They can be attributed to the distribution of NCC fillers in the structure of the WPI matrix network and the interactions of functional groups that reduced the space of biopolymer for the distribution and reaction that occur with water molecules [23,42].

#### 3.1.4. Water Solubility

The WS values in Table 4 show that the pure WPI film had the highest value of 92.174%, while the ones loaded with NCC had lower WS. NCC loading to the WPI film significantly reduced the WS by 26.719% (*p* < 0.05). Glycerol addition as the plasticiser was could increase the flexibility of the films and the hydrophilicity of the protein. It was required because of the presence of polar peptides that led to the high solubility of protein films in water [34]. The reduced WS of the film may be associated with forming a strong structure and bonding of the compounds added to the biopolymer matrix [23]. Moreover, the NCC filler’s dimension ratio and crystalline area have been reported as effective factors in the water-resistance characteristics of the biocomposite films [14,43].

#### 3.1.5. Moisture Absorption

Moisture absorption is one of the critical water-resistance characteristics in biocomposite films. In this study, the moisture absorptions of the WPI films are summarised in Table 4. The results show that the water absorption for the NCC-containing films decreased significantly (*p* < 0.05). The pure WPI film had a 5.1% higher water absorption value than those with NCC (*p* < 0.05), while the WPI film with 10% NCC had the least moisture absorption of 1.6% (*p* < 0.05). They can be attributed to the hydrophilicity of the WPI and the cellulose and the water–glycerol interactions [30]. It suggests that the addition of NCC fillers on the WPI film could prevent water molecule absorption and develop a biopolymer matrix with water-resistance property, as highlighted in earlier reports [14,23].

### 3.2. Mechanical Properties

**Figure 2:**
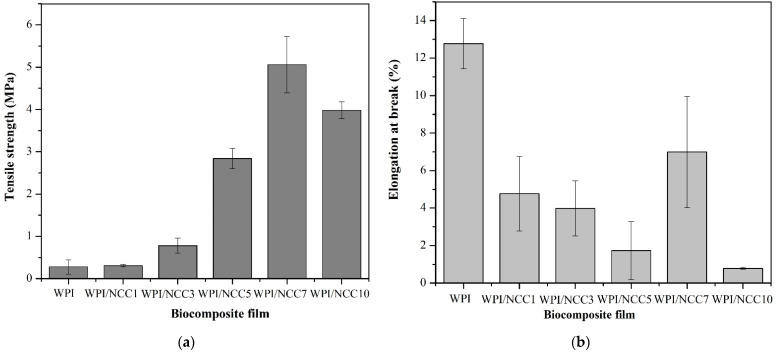
Mechanical properties of the biocomposite WPI film: (**a**) tensile strength and (**b**) elongation at break.

High mechanical properties of the film are required in food packaging to maintain them from stretching during usage. The tensile strength and elongation at the break of the prepared WPI films are shown in Figure 2. The tensile strength and elongation at break value for the WPI pure film were 0.281 MPa and 12.769%, respectively. Figure 2 shows a significant increase in the tensile strength of the WPI films containing up to 7% NCC (*p* < 0.05). The highest tensile strength of 5.060 MPa was obtained for the WPI film containing 7% of NCC. This was a significant increase, compared with the pure WPI film. The improvements in the tensile strength may be associated with the natural strength and chain stiffness of the NCC fillers, the high intra- and inter-molecular hydrogen bonds between NCC molecules and protein network, and the high compatibility between the fillers and the biopolymer matrix, as also suggested by others [14,41]. However, the tensile strength obtained in the WPI film containing 10% NCC decreased to 3.981 MPa. This decrease may be associated with the possible aggregation of NCC particles and their non-uniform distribution in the biopolymer matrix, as also reported earlier [14,16]. Similar findings were also reported by Reis et al. [7]. Thermoplastic starch/poly(butylene adipate-*co*-terephthalate with 1% of microcrystalline cellulose) showed higher tensile strength than the film without microcrystalline cellulose. However, a high microcrystalline content at 3% and 5% decreased the tensile strength. It was due to the weak interfacial bonding between the chain in polysaccharides and microcrystalline cellulose, weakening the strength of the biocomposite film. The highest strength achieved in this study (5.060 MPa) with the addition of 7% NCC was higher than that reported by Ekielski et al. [44] but was still lower than the one developed by Zhao et al. [16]. This difference could be explained by the differences in the molecular weight, type of filler, and the fibre sources of the developed NCCs in this study.

The elongation property of the WPI films containing NCC significantly decreased from 12.769% to 0.782%, with the increasing concentration of NCC from 0 to 10%, except for the 7% NCC (*p* < 0.05). This situation occurred because the loading of soft biopolymers such as WPI with stiff NCC fillers resulted in higher stiffness and had a brittle effect on the resulting film, compared with the pure WPI film [45].

The peaking of the elongation at break value for the 7% NCC loading can be explained as follows: It is related to the limited mobility and elasticity of the protein network due to the strong interaction between the filler and the biopolymer network [46]. The increase in tensile strength and the decrease in elongation at break value was also reported earlier that had the same results on the addition of NCC filler in film composites. The same trend was found from the research on soy protein isolate nanocomposites [16], alginate nanocomposites [46], the protein isolate nanocomposites with the addition of cellulose and rosemary essential oils [14], and the WPI composite film loaded with oat-based NCC [41].

### 3.3. Morphological Properties

#### 3.3.1. Morphology

**Figure 3:**
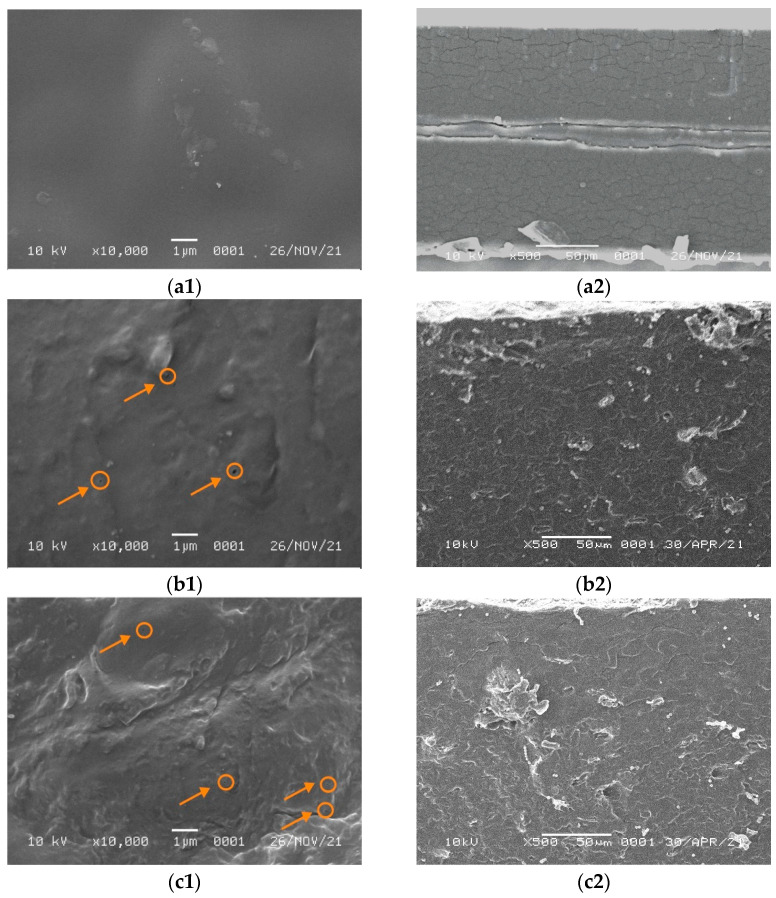
SEM micrographs of (**a1**) WPI on the surface, (**a2**) WPI on the cross section, (**b1**) WPI/NCC 5% on the surface, (**b2**) WPI/NCC 5% on the cross section, (**c1**) WPI/NCC 7% on the surface and (**c2**) WPI/NCC 7% on the cross section.

The obtained SEM micrographs of the pure WPI film surface containing 5 and 7% NCC are shown in Figure 3. They show that the morphology of the pure WPI film was homogeneous and smooth without any cracks and aggregates. On the other hand, the addition of NCC filler turned the surface rougher and heterogeneous with small aggregates in the film surface. Figure 3 shows that for the WPI film containing 5% NCC, the fillers were scattered with the formation of small voids (circle mark in Figure 3b1,c1) and agglomerates.

The filler dispersion was not homogenous for the WPI film containing 7% NCC and contained many agglomerates and voids covered on the surface. It probably occurred due to the WPI denaturation. The presence of NaOH and NCC filler in suspension was susceptible to aggregation because of the removal of the surface sulphate group [41]. The increase in the concentration of NCC filler promoted interaction between NCC and biopolymer forming aggregation due to the high interfacial area, surface energy, and NaOH in the film solution, as reported elsewhere [14,41]. In this case, the film matrix was severely damaged. A similar finding was obtained by Qazanfarzadeh et al. [41] and El wakil et al. [47] using oat-based NCC and bagasse pulp-based NCC loaded in WPI, which showed a non-uniform surface and non-homogenous agglomeration at high filler loadings. Furthermore, the surface of WPI film with NCC showed small voids or bubbles that might be formed during the drying process because of the trapped air and also as NCC filler acted as nucleating sites for voids growing [22].

#### 3.3.2. Surface Chemistry

**Figure 4:**
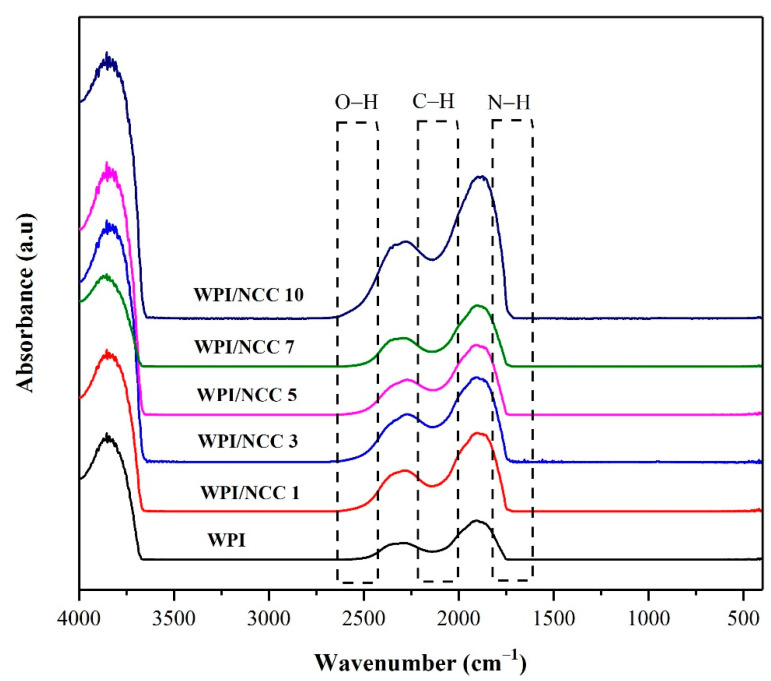
FTIR Spectra of whey protein film combined with nanocrystalline cellulose.

The identification of functional groups of the pure WPI film and the ones containing NCC were examined using FTIR analysis. FTIR is a spectroscopic method that has been widely used to analyse the IR spectrum of absorption in the biocomposite film. The IR spectra illustrated in Figure 4, showed various peak characteristics in all samples. The peaks at 2000–2500 cm^−1^ were caused by the vibration and bonding of the inter-hydroxyl and intra-hydroxyl groups O–H and C–H [14]. The peak at 2500 cm^−1^ for the pure WPI film slightly shifted to a higher wavenumber from 2500 to 2300 cm^−1^, depending on NCC loading. This shift can be linked to the vibration of the O–H bonds of NCC. The intensity of the O–H bond peak increased due to the formation of hydrogen bond between the –OH group of the WPI and the NCC in the polymer matrix [46]. The characteristic peak at around 2139 cm^−1^ could be attributed to the stretching of the C–H bond. Meanwhile, the peak that appeared around 1600 cm^−1^ was caused by vibration and stretching of the peptide bond formation conjugated by the amine group (NH) and acetone [48]. The overall results obtained from the IR spectra are slightly similar to the previous study of alginate film with NCC [46]. In this regard, the biocomposite film FTIR spectrum showed slightly the effect of NCC on the intensity of the spectra and changed the intramolecular interaction of the matrix.

**Figure 5:**
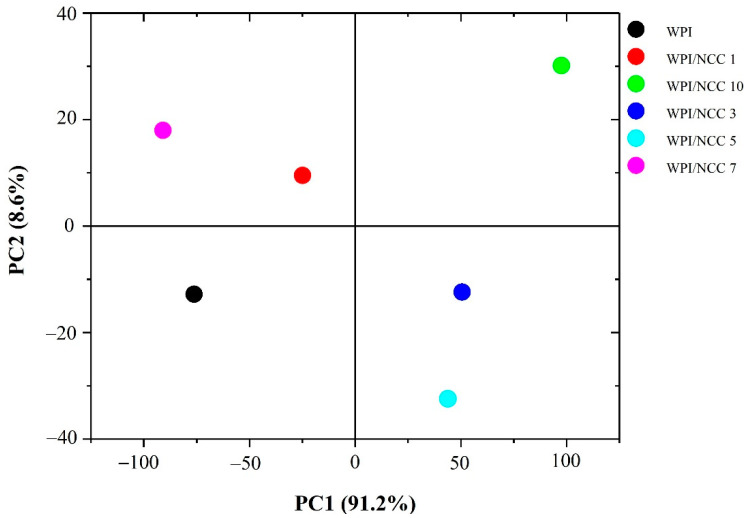
Scatterplot based on principal component analysis (PCA) of FTIR spectra of film.

In an extended analysis, the effect of NCC incorporation into WPI film was established using PCA on FTIR spectra data. The score plot for principal components PC1 and PC2 in the PCA model corresponded to the WPI with various NCC loading, as shown in Figure 5. Data values of FTIR spectra were distinctive. The scatter plot of PC1 and PC2 explained 97.6% of variability among samples. The PC1 axis mostly distinguished WPI/NCC 3, WPI/NCC 5, and WPI/NCC 10 on the positive side of PC1 from WPI, WPI/NCC 1, and WPI/NCC 7, which scattered on the negative side of PC1 while also being spread along in the PC2 axis. As shown in Figure 4, the positive influence of PC1 loading had slightly widened gradually in the spectra. The difference in the correlation indicated the slight effect of the NCC on the WPI films.

#### 3.3.3. Thermogravimetric Analysis

**Figure 6:**
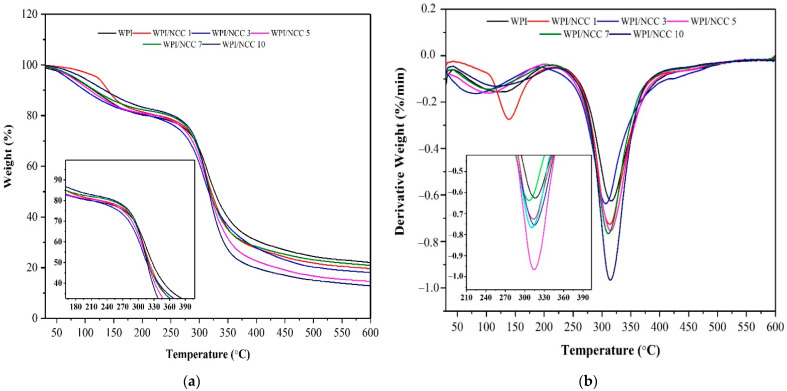
(**a**) TGA and (**b**) DTG biocomposite film.

Thermal properties of the pure WPI films and the ones containing NCC were analysed using TGA. One of the objectives of mixing the NCC fillers to the film was to evaluate the effect of NCC on the thermal stability of the resulting WPI-based film. The TGA curves for the pure WPI film and ones containing NCC are shown in Figure 6. The thermal value of films in Table 5 shows the thermal parameters such as T_onset_ (the temperature where weight loss started), T_10%_ (10% film weight loss temperature), T_50%_ (50% film weight loss temperature), and T_max_ (maximum degradation temperature).

The weight decreased for all films occurred in the temperature range of 200–400 °C. The onset temperature of the film showed an increase from 261 °C to 282 °C after the NCC loading. However, the maximum temperature of this film decreased slightly during the thermal analysis, while the WPI film containing 3% and 10% NCC had higher maximum temperatures than the rest. These results demonstrated the strengthening effect and contribution of NCC to WPI films.

The improvement in the thermal properties of the WPI-based films was caused by the increase in hydrogen bonding and decreased chain mobility owing to the addition of NCC biopolymers, as reported earlier [46,49,50]. Compared with the pure WPI film, T_onset_ and T_max_ of the NCC-containing films increased by 1–10 °C, respectively. Improvement of the film’s thermal stability could only be achieved for the NCC loading of >3%. In addition, the results show that the better thermal stability of the film containing NCC suggested a strong interaction between NCC and WPI matrix as biopolymers, a finding also reported elsewhere [46].

## 4. Conclusions

Overall findings demonstrated that the addition of NCC affected the properties of the resulting WPI films. The physical properties of the films decreased as the NCC concentrations increased. The properties of the films, such as WS and mechanical properties, were improved by additions of 5% and 7% of NCC. The FTIR spectra analysis indicated that NCC loading did not change the functional groups of the resulting WPI films. However, the PCA analysis showed a slight effect of NCC on the WPI film. On the other hand, the SEM analysis showed a significant morphological change for the WPI films containing 5% and 7% of NCC. The thermal stability of the films was enhanced by NCC loading, as shown by the increase in T_max_. Overall results concluded that loading PCL-based NCC as green reinforcement into WPI-based film could improve the properties of the resulting film and offered great potential for application as an alternative packaging film material in the industries. In these cases, further research is also needed to determine biocomposite film’s effectiveness in extending the application as food packaging.

## Figures and Tables

**Table 1:** The primary type of biopolymer in the development of packaging materials.

Biopolymer	Advantage Properties	Disadvantage Properties	Application	References
Starch	Good barrier against oxygen and good elongation	Poor mechanical and water barrier properties	Food packaging, biomaterial, composite film	[3,7]
Cellulose	Non-toxicity, biodegradability, and chemically stable	Poor water barrier properties and limited soluble with solvent	Packaging and biodegradable film	[8,9]
chitosan	Non-toxicity, good compatible and good film-forming	Poor barrier properties and insoluble in water	Antimicrobial agent, hybrid composite film	[10,11,12,13]
Whey protein	Excellent oxygen barrier, biodegradability, and good film-forming	Poor mechanical properties and high sensitivity to humidity	Biocomposite film, hybrid composite film	[14,15,16]
PLA	Biodegradability, good physical and mechanical properties	Low thermal properties	Food packaging and biomaterial	[9,17]

**Table 2:** Thicknesses data of whey protein film combined with nanocrystalline cellulose.

Film	Thickness (mm)
WPI	0.081 ± 0.02 ^ab^
WPI/NCC 1%	0.112 ± 0.01 ^d^
WPI/NCC 3%	0.117 ± 0.01 ^d^
WPI/NCC 5%	0.066 ± 0.01 ^a^
WPI/NCC 7%	0.086 ± 0.02 ^ab^
WPI/NCC 10%	0.091 ± 0.01 ^c^

WPI: whey protein isolate, NCC: nanocrystalline cellulose. The statistical analysis reported was based on ANOVA test. Different letters (a–d) in the same column indicate a significant difference (*p* < 0.05) among the means obtained by applying the Duncan’s test.

**Table 3:** Transparency of whey protein film combined with nanocrystalline cellulose.

Film	Wavelength (nm)					
	300	400	500	600	700	800
WPI	0	5.529 ± 0.10	10.803 ± 0.10	13.537 ± 0.09	15.417 ± 0.11	15.844 ± 0.46
WPI/NCC 1%	0	1.354 ± 0.09	2.160 ± 0.11	2.661 ± 0.85	2.941 ± 0.70	3.198 ± 0.70
WPI/NCC 3%	0	0.939 ± 0.58	2.136 ± 0.30	2.990 ± 0.00	3.479 ± 0.02	3.845 ± 0.11
WPI/NCC 5%	0	0.927 ± 0.00	2.380 ± 0.45	3.759 ± 0.23	4.833 ± 0.15	5.737 ± 0.15
WPI/NCC 7%	0	0.854 ± 0.07	1.989 ± 0.16	2.783 ± 0.31	3.259 ± 0.09	3.613 ± 0.39
WPI/NCC 10%	0	0.781 ± 0.09	1.330 ± 0.12	1.794 ± 0.11	2.062 ± 0.35	2.258 ± 0.26

WPI: whey protein isolate, NCC: nanocrystalline cellulose. The statistical analysis reported was based on ANOVA test.

**Table 4:** The water barrier properties of whey protein film combined with nanocrystalline cellulose.

Film	Moisture Content (%)	Water Solubility (%)	Moisture Absorption (%)
WPI	0.050 ± 0.02 ^c^	92.174 ± 5.48 ^d^	5.051 ± 0.31 ^d^
WPI/NCC 1%	0.046 ± 0.01 ^bc^	88.245 ± 1.60 ^d^	5.263 ± 0.17 ^d^
WPI/NCC 3%	0.032 ± 0.00 ^ab^	83.750 ± 3.68 ^cd^	4.310 ± 0.19 ^c^
WPI/NCC 5%	0.028 ± 0.00 ^ab^	82.353 ± 5.20 ^c^	4.082 ± 0.09 ^c^
WPI/NCC 7%	0.027 ± 0.01 ^ab^	75.806 ± 2.72 ^b^	2.889 ± 0.89 ^b^
WPI/NCC 10%	0.180 ± 0.01 ^a^	65.455 ± 1.78 ^a^	1.695 ± 0.16 ^a^

WPI: whey protein isolate, NCC: nanocrystalline cellulose. The statistical analysis reported was based on ANOVA test. Different letters (a–d) in the same column indicate a significant difference (*p* < 0.05) among the means obtained by the application of the Duncan’s test.

**Table 5:** Thermal value of whey protein film combined with nanocrystalline cellulose.

Film	T_onset_ (°C)	T_10%_ (°C)	T_50%_ (°C)	T_max_ (°C)
WPI	261	294	348	352
WPI/NCC 1%	265	297	340	350
WPI/NCC 3%	268	295	348	356
WPI/NCC 5%	273	297	335	355
WPI/NCC 7%	273	297	339	347
WPI/NCC 10%	282	302	331	348

WPI: whey protein isolate, NCC: nanocrystalline cellulose.

## Data Availability

The data presented in this study are available on request from the corresponding author.
